# A Systematic Review of Sporadic Creutzfeldt-Jakob Disease: Pathogenesis, Diagnosis, and Therapeutic Attempts

**DOI:** 10.3390/neurolint16050079

**Published:** 2024-09-20

**Authors:** Maria Carolina Jurcau, Anamaria Jurcau, Razvan Gabriel Diaconu, Vlad Octavian Hogea, Vharoon Sharma Nunkoo

**Affiliations:** 1 Faculty of Medicine and Pharmacy, University of Oradea, 410087 Oradea, Romania; mariacarolina.jurcau@student.uoradea.ro (M.C.J.);; 2 Department of Psycho-Neurosciences and Rehabilitation, University of Oradea, 410087 Oradea, Romania; 3 Neurorehabilitation Ward, Clinical Emergency County Hospital Bihor, 410169 Oradea, Romania

**Keywords:** Creutzfeldt-Jakob disease, prions, MRI, PMCA, RT-QuIC, WHO and EUROCJD diagnostic criteria, treatment

## Abstract

Creutzfeldt-Jakob disease is a rare neurodegenerative and invariably fatal disease with a fulminant course once the first clinical symptoms emerge. Its incidence appears to be rising, although the increasing figures may be related to the improved diagnostic tools. Due to the highly variable clinical picture at onset, many specialty physicians should be aware of this disease and refer the patient to a neurologist for complete evaluation. The diagnostic criteria have been changed based on the considerable progress made in research on the pathogenesis and on the identification of reliable biomarkers. Moreover, accumulated knowledge on pathogenesis led to the identification of a series of possible therapeutic targets, although, given the low incidence and very rapid course, the evaluation of safety and efficacy of these therapeutic strategies is challenging.

## 1. Introduction

Creutzfeldt-Jakob disease (CJD), first described in 1920 by German neurologist Hans Gerhard Creutzfeldt and shortly afterward by Alfons Maria Jakob [1], is a rare, rapidly progressive, and invariably fatal neurodegenerative disease [2]. It entered the spotlight in the 1990s when the outbreak of the bovine spongiform encephalopathy and the variant CJD transmitted to humans led to the initiation of international surveillance programs for CJD worldwide [3,4,5].

The annual incidence is around 1–2/1 million inhabitants but increases with age [2]. However, national surveillance studies drew attention to an increase in the incidence of prion diseases, although it is not clear whether this reflects an actual increase in the incidence of the diseases or increased awareness of physicians and improved availability of diagnostic tools [6,7]. Moreover, recent case reports linked the onset of CJD to infections with SARS-CoV-2 or vaccination against COVID-19 [8,9].

The disease belongs to a group of neurodegenerative conditions affecting humans and animals that include sporadic and genetic Creutzfeldt-Jakob disease, sporadic (considered a subtype of sporadic CJD) and familial fatal insomnia, Gerstmann-Sträussler-Scheinker disease, Kuru disease and variant CJD in humans; bovine spongiform encephalopathy (“mad cow disease”) in cattle; scrapie in sheep; and the chronic wasting disease in mule deer and elk. The etiology of these diseases was elusive for many years and subject to diverse hypotheses. Fragments of nucleic acids, polysaccharides, or a protein have all been discussed as possible etiological agents, until Stanley Prusiner introduced the term “prion” to name the infectious proteinaceous particle and detailed the protein hypothesis [10,11], which, although initially greeted with skepticism, allowed him to win the Nobel Prize in Physiology or Medicine in 1997.

For a long time, the diagnosis was possible only post-mortem by showing the spongiform modifications induced in the brain, but in recent years, more sophisticated laboratory evaluations and brain imaging studies can aid in diagnosing the disease earlier [12]. Nonetheless, the diagnosis is often delayed by several months from the onset of the first symptoms [13], much to the distress of patients, who witness the rapid decline of their health and of their family members.

In our manuscript, we review the pathophysiology, clinical picture, diagnostic criteria, and differential diagnosis of CJD, highlighting recent therapeutic strategies evaluated in preclinical studies.

## 2. Sporadic Creutzfeldt-Jakob Disease—Overview of the Current Knowledge on Etiology, Pathogenesis, and Clinical Picture

### 2.1. Etiology

Currently, the etiology of CJD is considered a prion, or proteinaceous infectious particle (PrP), that takes on conformational changes and tends to spread in the central nervous system (CNS), leading to histopathologic changes.

The protein PrP is normally produced by all vertebrates cells and is highly conserved in mammals [14]. The human PrP gene (*PRNP*) is found on chromosome 20 and encodes a 253 amino acid protein. After removal of the C- and N-terminal signal peptides, the mature 208 amino acid protein (PrP23-231) is guided by chaperones into the endoplasmic reticulum and Golgi apparatus to fold and undergo post-translational modifications such as attachments of glycans or the addition of a glycophosphatidylinositol anchor to the C-terminus [15]. The cellular prion protein (PrP^C^) is expressed in most tissues of the body but mostly in the CNS, particularly in neurons [16]. Its functions are still elusive [17]; roles in cell adhesion and signaling, in copper metabolism [18], or the binding and transport of nucleic acids have been suggested [19]. Recently, it has been shown that PrP^C^ interacts with and modulates a large number of membrane receptors (especially glutamate receptors), ion channels, and amino acid transporters [20,21]. Due to various reasons, the normal host protein undergoes a templated polymerization, igniting a series of pathogenic cascades and activating astrocytes and microglia, culminating in histopathological spongiform changes and rapid neuronal loss.

### 2.2. Pathogenesis

The key events triggering PrP^Sc^ formation are still incompletely elucidated. External factors (oxidative stress, inflammation, or age), as well as pathogenic *PRNP* mutations, may all be involved [22], followed by a failure of the cellular proteostasis machinery in clearing the misfolded protein [23]. Nuclear magnetic resonance imaging studies in recombinant mouse PrP protein have shown that normal cellular PrP is structurally about 40% alpha helix and 3% beta-sheet, while analyses performed on the altered protein from sheep with scrapie revealed a significantly altered conformation, with 45% beta-sheet and 30% alpha helix [24], making the aggregated protein resistant to enzymatic digestion. Genetic factors (several polymorphisms of the *PRNP* gene) might promote the conversion of PrP^C^ into PrP^Sc^ [25].

In the case of orally or transcutaneously transmitted prion diseases, it is still debated how prions reach the CNS. One possibility would be hematogenous spread via immune cells and lymphoid organs [26], as demonstrated by the transmission of bovine spongiform encephalopathy by blood transfusion in sheep [27]. PrP^C^ is expressed on cell surfaces of T lymphocytes [28], macrophages, natural killer cells, regulatory T cells, and dendritic cells [29] and has important roles in the organization of lymphoid tissues, such as lymph nodes and the spleen [30]. When PrP^Sc^ infects the host, it uses the immune cells to replicate, converts PrP^C^ to PrP^Sc^, and accumulates [29,31]; after which, it spreads to the CNS via B cells [32], follicular dendritic cells [33], or other dendritic cell populations [34].

Another route for reaching the CNS would be from the gut via the parasympathetic fibers of the vagus nerve [35] following modifications of the gut microbiota, similar to Parkinson’s disease, where the abnormally folded α-synuclein may reach the CNS via parasympathetic fibers and spread in the CNS in a prion-like fashion [36].

Alternatively, infections with neurotropic strains of viruses, such as influenza viruses [37], SARS-CoV-2 [8,38], or vaccination against COVID-19 [9], may induce conformational changes in PrP^C^ to form PrP^Sc^, which then spreads to neighboring neurons through different mechanisms such as exosomes [39], receptor-mediated internalization [40], or nanotubes [41]. However, the expression of PrP^C^ is required for prion formation and replication, as shown by the resistance to the disease of mice devoid of PrP^C^ [42].

Growing evidence points toward seeding nucleation as the mechanism leading to prion aggregation, during which, PrP^Sc^ oligomers convert PrP^C^ monomers and incorporate them into protofibrils and fibrils [43]. Fibril fragmentation can create new nucleation sites and, by incorporating new PrP^C^ monomers, amplify the reaction [44]. PrP^C^ being anchored by glycophosphatidylinositol (GPI) in the lipid rafts of the cellular membranes can act as a receptor and concentrate PrP^Sc^ molecules on cell surfaces, thereby compromising the cellular integrity and inducing neurotoxic effects [45]. In addition, during the conversion phase, PrP^C^ twists along one side of the fibril while being membrane-anchored by GPI, leading to membrane distortion and cellular damage [46]. Internalized PrP^Sc^ aggregates can block the ubiquitin–proteasome system and activate the unfolded protein response signaling pathway [47], leading to impaired protein synthesis, with consequent synaptic dysfunction and neuronal loss, as well as the deacetylation and degradation of PIKfyve (a FYVE finger-containing phosphoinositide kinase), resulting in a reduction of phosphoinositide diphosphate levels. The latter causes impairment in the maturation of endosomes, leading to enlarged endosomes that may become intracellular vacuoles [48]. Following shedding from the plasma membrane, it is cleaved by ADAM10 (A disintegrin and metalloproteinase domain-containing protein 10), resulting in N- and C-terminal fragments that retain biological activity [25].

Both neurons and glial cells are involved in the pathological processes. Microglial cells attempt to clear PrP^Sc^ but also release inflammatory cytokines that ignite neuronal apoptosis [15], while astroglia is also activated and polarizes to the A1 neurotoxic phenotype, supplementally contributing to neuronal damage [49]. Even oligodendrocytes exhibit impaired energy metabolism and solute transport caused by altered gene transcription, as shown by the significantly reduced levels of mRNAs for galactosylceramidase, monocarboxylic acid transporter 1, and solute carrier family 2 member 1: glucose transporter member 1: GLUT1 [50].

### 2.3. Classification

Creutzfeldt-Jakob disease can be classified into [15]:

- sporadic CJD, accounting for more than 80% of cases, thought to be due to unknown cellular events that lead to the conversion of PrP^C^ to PrP^Sc^,
- genetic CJD, about 10–15% of cases, linked to a series of mutations in the PRNP gene transmitted in an autosomal-dominant pattern with variable penetrance [25], and
- acquired CJD, caused by human-to-human transmission via surgical or medical procedures (corneal transplants, dural grafts, administration of human pituitary-derived gonadotrophins, and human-derived growth hormone) [51,52,53].

Another classification, at the histo-molecular level is based on the *PRNP* polymorphism at codon 129 (methionine—M or valine—V) and the physicochemical properties of the PrP^Sc^ strain after limited proteinase K digestion (type 1 cleaved at residue 82 and migrating at 21 kDa and type 2 cleaved at residue 97 and having a molecular mass of 19 kDa) [54,55]. Initially, six molecular groups were identified, each correlating with clinical and neuropathological phenotypes of sporadic CJD: MM1, MV1, VV1, MM2, MV2, and VV2. Subsequently, the MM2 group has been separated into a “cortical” and a “thalamic” variant (MM2C and MM2T, respectively), while, in the MV2 group, a subtype with cerebellar kuru-type amyloid plaques, termed the MV2C subtype, has been described [56]. A MM1 subtype showing widespread PrP-amyloid plaques in the white matter has been designated as p-MM1 [57], and because MM1 and MV1 are phenotypically virtually indistinguishable, they were merged into the MM(V)1 subtype [58]. MM1, VV2, and MV2 account for the large majority of cases [58]. Interestingly, PrP^Sc^ types 1 and 2 have been found to co-occur in the same brain specimens in about one-third of cases [58,59].

### 2.4. Neuropathology

The microscopic changes described in CJD consist of depositions of PrP^Sc^ aggregates, microglial activation, spongiform degeneration affecting various areas of the brain, neuronal loss, and astrocytic gliosis [58].

#### 2.4.1. PrP^Sc^ Depositions

PrP^Sc^ deposits can be detected either isolated or in combination, due to the phenotypic heterogeneity of the disease.

1. Synaptic deposits are the most common ones, consisting of microgranular, diffuse PrP^Sc^ deposits identified in the cerebral cortex (mainly the occipital one) and in the molecular layer of the cerebellum.
2. Plaque-like deposits are rounded and well defined, described in the cerebral cortex, striatum, thalamus, granular layer of the cerebellar cortex, and cerebellar white matter, as well as, occasionally, in the cerebral white matter.
3. Perivacuolar deposits are found in the cerebral cortex, associated with large, confluent vacuoles.
4. Perineuronal deposits are seen in the pyramidal neurons of the fifth cortical layer and in the hippocampus, delineating the perikarya and dendrites of neurons.

#### 2.4.2. Microglial Activation

Microglia are activated early in the disease course, attempting to clear the accumulated PrP^Sc^ but also favors neurodegeneration via neuroinflammation. The finding of numerous activated microglia resulting from the proliferation of resident cells early in the disease course in animal models was confirmed in human cases as well by showing widespread microgliosis in post-mortem sporadic CJD brains [60].

#### 2.4.3. Spongiform Degeneration

Spongiform changes are characteristic features of CJD, although the vacuole size and distribution may vary, depending on disease subtype and stage. Three types of spongiform changes can be recognized [58]:

1. Small, round vacuoles 2–5 μm in diameter, located in the cerebral cortex, striatum, thalamus, and molecular layer of the cerebellar cortex, are most commonly found.
2. Large vacuoles, 15–20 μm in diameter, with a tendency to merge into grape-like structures, are often observed in the cerebral cortex.
3. Vacuoles of intermediate size are often seen in subcortical structures and the cerebellum.

#### 2.4.4. Synaptic Loss

Normally, PrP^C^ is concentrated in synaptic terminals, where it co-localizes with synaptophysin. As such, the conversion of PrP^C^ into PrP^Sc^ contributes to synaptic dysfunction and loss. An increase in synaptic proteins, such as SNAP25, Rab3A, synaptotagmin 1, or clathrin, as a consequence of impaired synaptic vesicle release and re-uptake, has been convincingly demonstrated in CJD by several researchers [61,62].

#### 2.4.5. Amyloid Plaques

About 10–15% of CJD patients have also compact amyloid plaques formed by PrP^Sc^ aggregates, located mainly in the cerebellar cortex, confined between the granular and Purkinje cell layers, although, in cases with a more prolonged disease course, these plaques may be found in the cerebral cortex as well [58]. They measure up to 30 μm, have a dense core surrounded by thin, amyloid radial bundles [63], and mainly characterize the MV2 subtype [64].

#### 2.4.6. Secondary Tauopathy

A deposition of abnormal hyperphosphorylated tau aggregates has been documented in CJD as well, appearing as small, punctate, neuritic deposits (in about 16% of cases) or also associating with neuronal and glial tau pathology (84% of cases) [65]. Most often, tau pathology affects the VV2 and MV2K subtypes [66].

#### 2.4.7. Astrocytic Gliosis

PrP^Sc^ accumulates in astrocytes early in the disease course, leading to their polarization towards the A1 phenotype. Reactive astrocytes have been found mainly in white matter but also in the thalamus, hypothalamus, and cerebellum of human CJD patients [67]. However, the findings of increased mRNA levels of markers of neuroprotective A2 astrocytes in the thalamus and hippocampus and of increased mRNAs for A1-specific markers in the hippocampus and cortex of prion-infected mouse brains suggest a heterogenous astrocytic response across different brain regions in prion diseases [68]. Moreover, different prion strains were associated with distinct patterns of astrocytic activation [69].

Research has shown that astrocytic activation precedes microglial activation and neurotransmission-associated disturbances [70], suggesting that, in the early stages of the disease, neuroprotective glial phenotypes prevail, while, in the later stages of prion infection, the neurotoxic ones are more widespread [71].

Although the role of glial cells in prion diseases is not yet fully understood and is the subject of active research [72], astrogliosis and neuronal loss are both observed in CJD, similar to other neurodegenerative diseases [73].

### 2.5. Clinical Picture

The diagnosis of sporadic CJD is challenging, because patients present with a wide range of non-specific symptoms and signs, but the disease is known as rapidly progressing dementia-associating visual and cerebellar signs, myoclonus, the patients develop rapidly akinetic mutism, and the mean survival is about 6 months [58].

The prodromal phase is usually overlooked, because patients exhibit behavioral changes, unspecified fear, eating abnormalities and weight loss, depressive mood changes, or may complain of sleeping disturbances [74,75].

Patients seek medical advice usually when cognitive functions start to decline, in the early stage of CJD. Cognitive impairment usually affects all domains. In a relatively frequent phenotype, previously known as the Heidenhain variant of CJD, visual symptoms are the presenting symptoms and consist of the blurring of vision and diplopia, later associated with complex visual hallucinations and progressing to cortical blindness [76]. In the phenotype known as the Brownell-Oppenheimer variant, cerebellar ataxia (mainly gait ataxia) appears first and may be the only neurological sign for several weeks [77]. More rarely, the first neurological signs may be a parkinsonian syndrome [78], hemiparesis mimicking a stroke [79], various types of epileptic seizures [80], or myoclonus, although the latter is considered to be a rather characteristic sign in CJD in later stages [81].

The disease progresses rapidly to significant cognitive impairment, associating other neurological signs with the presenting ones, such as dysarthria or loss of speech, and patients rather rapidly develop akinetic mutism [58]. Death occurs within weeks to months, although, in rare cases, survival may be possible for a few years [82].

With the identification of the molecular subtypes of CJD, it became clear that these subtypes have rather characteristic phenotypic presentations, pathology, and mean survival times, as shown in Table 1.

### 2.6. Evaluation

#### 2.6.1. Laboratory Studies

Initial workup should include laboratory tests for dementia, such as serum chemistry panel, liver enzymes, ammonium levels, complete blood count, erythrocyte sedimentation rate (with blood cultures if any infection suspected), evaluation of the thyroid function, tests for neurosyphilis, and measurement of the B12 and folate levels, as well as serum levels of anti-thyroperoxidase antibodies to rule out Hashimoto encephalopathy.

If the patient’s history or general status and symptoms might be compatible with a paraneoplastic syndrome, specific antibodies should be searched (as detailed in the Section 2.8.

Routine CSF analysis is usually unrevealing, showing only a mild increase in the total protein levels in about one-third of cases [58].

A series of protein markers identified in the CSF have rather high sensitivity and specificity, being valuable aids in the diagnosis of CJD:

- The family of 14-3-3 proteins was the first CSF biomarker to be used in diagnosing CJD. They are cytosolic proteins with regulatory functions, released into the CSF during neuronal damage. Although they may be increased in other conditions associated with neuronal destruction as well, the increase in 14-3-3 protein in the CSF shows an 87% sensitivity and 66% specificity if assessed semi-quantitatively by Western blotting, while assessment with ELISA increases the specificity to 84% [84].
- The microtubule-associated protein tau (total tau—t-tau) as a marker of neuroaxonal degeneration is also largely used as a surrogate marker for the pre-mortem diagnosis of CJD [58,84], although it also increases in Alzheimer’s disease (AD) [85]. The cut-offs for differential diagnosis between CJD and AD are still not set, ranging between >1072 pg/mL [86] and >1583 pg/mL [87]. Because phosphorylated tau (p-tau) shows a less prominent increase in the CSF in CJD as compared to AD, the t-tau/p-tau ratio (cut-off < 0.075) [88] adds to the sensitivity (around 96%) and specificity (98–100%) [88]. In addition, the t-tau levels may suggest specific subtypes (being highest in the MM1, MV1, and VV2 types) [89] and could provide information related to prognosis and life expectancy [90].
- Alpha-synuclein also shows significant increases, even more pronounced than in Parkinson’s disease or dementia with Lewy bodies [91] (proposed cut-off values being > 3300 pg/mL) [91], with a sensitivity of around 90% [92].
- Neurofilament light (Nfl), a small subunit of a membrane cytoskeleton polymer, increases significantly in several neurodegenerative diseases, CJD included [93]. The proposed cut-off is 7000 pg/mL [94], with a sensitivity of around 85% [92].
- YKL-40 (also known as chitinase 3-like 1), a marker of neuroinflammation, also shows an increase above the proposed cut-off of 315 ng/mL [95] in the CSF of patients with CJD, its sensitivity being around 76% [92], along with other markers of glial activation, such as CHIT-1 or GFAP (glial fibrillary acidic protein) [96,97].
- Other biomarkers have been studied in recent years and shown to increase in sporadic CJD, such as neurogranin (related to synaptic plasticity) [98], ubiquitin (a marker of neuritic damage and neuroinflammation) [99], and calmodulin [100], as well as mitochondrial malate dehydrogenase 1 (a marker of mitochondrial dysfunction and oxidative stress) [101].

Because the assessment of these fluid biomarkers is invasive, requiring a lumbar puncture, the value of plasma biomarkers has been studied, but, unfortunately, they have a lower diagnostic accuracy. Increased plasma tau has a higher diagnostic accuracy compared to increases in plasma Nfl [102], but both are inferior to the evaluation of CSF total tau and 14-3-3 protein. Plasma microRNA studies have emerged as complementary tests in recent years [103].

Cell-free conversion assays based on the self-replicating ability of PrP^Sc^ can significantly aid in the diagnosis of prion disease. At the beginning of the third millennium, the protein misfolding cyclic amplification (PMCA) technique was used [104], requiring the mixing of suspected infected material homogenates (as source of PrP^Sc^) with normal brain homogenate (source of PrP^C^), followed by repeated steps of incubation (allowing aggregate growth) and sonication (which fragmented the aggregates into smaller seeds to promote aggregate growth in the next incubation step). In the end, proteinase K (PK) treatment eliminated PrP^C^, and Western blotting revealed the residual PrP^Sc^ [105].

In 2008, a new technique, quaking-induced conversion (QuIC), was described [106], based on mixing bacterially expressed folded recombinant PrP with brain homogenates, followed by periods of orbital shaking and rest at controlled temperatures. Read-outs are performed by Western blotting after PK digestion. The method allowed for the detection of very low concentrations of PrP^Sc^ but was time-consuming.

A huge step forward in the early diagnosis of CJD was the development of the real-time quaking-induced conversion (RT-QuIC) assay, which indirectly detects PrP^Sc^ in the CSF, nasal fluids, or brain homogenate in concentrations as low as 10^−13^–10^−15^ g [107]. Supplementing the conversion medium with thioflavin T (ThT) allows for reading by using real-time ThT fluorescence measurements, which makes the assay faster and less expensive. The method has been integrated since 2017 into the International CJD Surveillance Network diagnostic criteria for CJD [108]. It has a high sensitivity (80–96%), although it tends to be lower in the MM1 subtype and the genetic forms of the disease, and a 99–100% specificity [105] but is expensive and less standardized compared to other surrogate biomarkers, such as 14-3-3 protein or t-tau [96].

RT-QuIC can also be performed in samples obtained from nasal brushing, which is less invasive than a lumbar puncture, with similar sensitivity and specificity (97% and 100%, respectively) [109]. However, since only preliminary data are available regarding the use of this diagnostic tool, and because a lumbar puncture would be needed for the screening of other neurodegenerative diseases, it is questionable whether RT-QuIC from nasal mucosa brushing samples will replace CSF RT-QuIC [96].

#### 2.6.2. Imagistic Studies

The preferred imaging modality for suspected CJD is a MRI. Initially, the signal abnormalities are subtle but become more pronounced as the disease progresses and is associated with rapidly progressing cerebral atrophy.

At first, due to the low sensitivity of conventional MRI sequences in detecting cortical signal abnormalities, only abnormal signals in the caudate and putamen were discussed as potential diagnostic features [110]. In 2009, cortical signal hyperintensities in at least two lobes, except the frontal lobes on diffusion-weighted imaging (DWI) and fluid-attenuated inversion recovery (FLAIR), were added to the diagnostic criteria [111], while, in 2020, Bizzi and coworkers proposed the inclusion of signal abnormalities in the frontal cortex as well [112] while pointing out that the cingulate gyrus, insula, hippocampus, and cerebellum should be excluded, because they can be spontaneously hyperintense on the DWI of healthy individuals.

Specifically, abnormal signals appear usually bilaterally, but sometimes asymmetrically, in the [112,113]:

- Cerebral cortex, affecting most commonly the insula, cingulate gyrus, or superior frontal gyrus; commonly the cuneus, precuneus, medial and/or inferior frontal gyri, occipital gyri, angular and supramarginal gyri, or superior parietal lobule (depending also on the clinical variant); and less commonly the medial and superior temporal gyri or the precentral and postcentral gyri
- Basal ganglia, most commonly in the putamen and striatum but also in the thalamus

Depending on the phenotypic variants, the first signs may be detected [113]:

- In the cerebellum, in the Brownell-Oppenheimer variant,
- In the parietooccipital cortex in the Heidenhain variant,
- In the putamen and thalamus in the Stern-Garcin variant, with extrapyramidal features from onset.

The most sensitive sequences are diffusion-weighted images (DWIs) [114], showing hyperintensities in the characteristic regions mentioned above, due to a combination of diffusion restriction (reduced diffusion of water caused by compartmentalization within vacuoles) or deposition of prion protein that restricts the free diffusion of water and to a high T2 signal that “shines through” to the DWI imaging (T2 shines through) [115]. The resultant signs are cortical ribboning, the pulvinar sign (hyperintensities in the thalamic pulvinar nuclei), and the hockey stick sign (hyperintensity of the medial thalamus) [116], previously thought to be more characteristic of the variant CJD. With disease progression, DWI signal intensity abnormalities may fade, likely due to neuronal loss and increased atrophy [117].

T2/FLAIR hyperintensities are more subtle than DWI changes and may be absent early in the disease course but are usually present in the same areas as DWI signal changes [115].

The ADC (apparent diffusion coefficient) aspect varies depending on the timing in the course of the disease. Initially, low values may be seen even before the DWI changes to be later replaced with normal intensity signals [115], probably related to neuronal loss and associated with prominent atrophy.

T1 sequences may, on occasion, show a high signal in the globus pallidus, while no abnormal enhancement can be detected after gadolinium administration [114].

The overall sensitivity of MRI abnormalities ranges between 69 and 92% [12,25,118], depending also on the expertise of the neuroradiologist. It can also suggest subtypes: basal ganglia hyperintensities are detected most often in VV2 and MV2 subtypes [12], while cortical ribboning characterizes mainly the MM subtypes [118].

#### 2.6.3. Electroencephalography (EEG)

The EEG abnormalities depend on the disease stage. Initially, there may be only a non-specific, diffuse slowing background, later replaced by the characteristic symmetrical, generalized, bi-, triphasic, or mixed periodic sharp-wave complexes occurring with a frequency of about 1 cycle/second that are included in the World Health Organization diagnostic criteria. However, these findings tend to disappear in the late stages of CJD [119]. Their sensitivity ranges between 64 and 67%, with a 74–94% specificity [120]. In addition, other EEG abnormalities, such as convulsive and non-convulsive status epilepticus, have been reported in the literature [121,122], in which the EEG aspect may be difficult to discriminate from CJD but which responds to antiepileptic medication [25].

#### 2.6.4. Positron Emission Tomography (PET) and Single Photon Emission Computed Tomography (SPECT)

PET using ^18^fluorine-fluorodeoxyglucose (^18^F-FDG) and other radiotracers can reveal cortical and subcortical hypometabolic areas and may suggest the presence of a specific CJD subtype but fails to differentiate between sporadic and genetic forms of CJD [123], while SPECT may identify hypoperfusion in the brain regions that exhibit DWI hyperintensities as well [124]. Although these methods could allow for further refinement of the imaging studies, they have not been incorporated into the diagnostic criteria and are just being explored.

### 2.7. Diagnostic Criteria

The World Health Organization proposed the following criteria for diagnosing sporadic Creutzfeldt-Jakob disease [125]:

(a) Possible CJD:
  - progressive dementia
  - EEG atypical or not known
  - duration <2 years
  - At least **two out of the following four** clinical features:
    - myoclonus
    - visual or cerebellar disturbance
    - pyramidal/extrapyramidal dysfunction
    - akinetic mutism
(b) Probable CJD: (in the absence of an alternative diagnosis from routine investigation)
  - progressive dementia
  - at least two of the following four clinical features:
    - myoclonus
    - visual or cerebellar disturbance
    - pyramidal/extrapyramidal dysfunction
    - akinetic mutism
  - a typical EEG, whatever the clinical duration of the disease
  - a positive 14-3-3 assay for CSF and a clinical duration to death <2 years
(c) Confirmed (definite) CJD:
  - neuropathological confirmation
  - confirmation of protease-resistant prion protein (PrP) (immunocytochemistry or Western blot)
  - Presence of scrapie-associated fibrils

The European and UK criteria have incorporated characteristic MRI findings since 2009 [111] and were further refined in the subsequent updated guidelines. Currently, the 2017 revised diagnostic criteria for sporadic Creutzfeldt-Jakob disease [126] define:

A. **Definite** sporadic CJD:
  Progressive neurological syndrome
  Neuropathologically **or** immunohistochemically **or** biochemically confirmed
B. **Probable** sporadic CJD:
  1. Rapidly progressive cognitive impairment
  Two of the following:
    - myoclonus
    - visual or cerebellar symptoms/signs
    - pyramidal or extrapyramidal features
    - akinetic mutism
  **and** a typical EEG showing **generalized periodic complexes**
  2. Rapidly progressive cognitive impairment
    Two of the following:
      - myoclonus
      - visual or cerebellar symptoms/signs
      - pyramidal or extrapyramidal features
      - akinetic mutism
  **and** a typical MRI brain scan, showing a high signal in the caudate/putamen or at least two cortical regions (temporal, parietal, or occipital) on either the DWI or FLAIR
  3. Rapidly progressive cognitive impairment
  Two of the following:
    - myoclonus
    - visual or cerebellar symptoms/signs
    - pyramidal or extrapyramidal features
    - akinetic mutism
  **and** positive CSF 14-3-3 protein
  **and** without routine investigations indicating an alternative diagnosis
  4. Progressive neurological syndrome and positive RT-QuIC in CSF or other tissues
C. **Possiblesporadic** CJD:
  Rapidly progressive cognitive impairment
  Two of the following:
    - myoclonus
    - visual or cerebellar symptoms/signs
    - pyramidal or extrapyramidal features
    - akinetic mutism

**and** duration < 2 years

**and** the absence of a positive result for any of the four tests that would classify the case as “probable”

**and** without routine investigations indicating an alternative diagnosis

These criteria have a 92% sensitivity for detecting probable sporadic CJD, rising to almost 98% when all investigations are performed [12]. However, not every patient can undergo extensive evaluation, and biomarker testing has inherent limitations [127], which is why alternative diagnoses should not be ruled out easily [128].

### 2.8. Differential Diagnosis

#### 2.8.1. Clinical Differential Diagnosis

Because the clinical presentation of CJD is quite heterogeneous, the differential diagnosis is exhaustive, and every effort should be made to identify the conditions mimicking CJD that are amenable to treatment.

(A) Other neurodegenerative diseases, such as Alzheimer’s disease, frontotemporal dementia, dementia with Lewy bodies, corticobasal degeneration, and multiple system atrophy, usually do not evolve as rapidly as CJD, although, on occasion, these conditions may exhibit an accelerated course. In addition, drug-related adverse events may lead to symptoms that might suggest CJD (tricyclic antidepressants or lithium leading to myoclonus and antiepileptics causing ataxia), or infections, metabolic disturbances, or various toxins may cause delirium in patients already cognitively impaired by an underlying neurodegenerative condition and mimic a rapid progression [129]. This is why a thorough history regarding recent changes in drugs and lab tests to monitor for metabolic disturbances or infections is advocated. Nevertheless, these conditions may be more similar to CJD than previously thought, since research has convincingly shown that the characteristic abnormally folded pathological protein propagates from cell to cell in a prion-like manner [36,130,131], and the possibility of iatrogenic transmission of Alzheimer’s diseases has been shown recently [132].

(B) Cerebrovascular conditions, either strategically placed stroke, multiple infarcts, or dural arteriovenous fistulas, and sinus thrombosis, as well as hypertensive encephalopathy, can cause rapidly progressing dementia, but usually, imaging may indicate the correct diagnosis.

(C) Infections of the central nervous system (herpes simplex encephalitis or Japanese encephalitis) usually occur in younger patients and are associated with fever and nuchal stiffness, as well as acute onset. Tertiary-stage neurosyphilis, with an increasing incidence in many developed countries [133], may also cause ataxia, movement disorders, and even psychosis, but serologic testing confirms the diagnosis. Lyme disease, caused by the *Borrelia burgdorferi* spirochete, also affects skin, joints, heart, and peripheral nervous system, aside from causing encephalomyelitis [129]. Opportunistic CNS infections in immunocompromised patients (toxoplasmosis in HIV-infected patients, cryptococcal meningitis, or progressive multifocal leukoencephalopathy caused by reactivation of the JC virus), as well as HIV-associated neurocognitive disorder, may also present as rapid progressive dementias in a considerable percentage of patients [129].

(D) Many toxic-metabolic etiologies of rapid progressive dementias are reversible if proper treatment is started early. Lithium may induce ataxia, tremor, myoclonus, or cognitive disturbances, even if the serum levels are within therapeutic ranges in patients with infections, dehydration, or renal impairment [134]. Lead poisoning, especially exposure to organic lead (gasoline additive), may adversely affect the limbic system and cause agitation, hallucinations, and altered sleep [135]. Thiamine deficiency (Wernicke-Korsakoff) syndrome should always be considered, not only in alcoholic patients but in every condition with the potential of causing malnutrition, such as gastric bypass surgery or repeated vomiting [136]. Some of the characteristic MRI findings may overlap those seen in CJD (detailed below), but urgent thiamine treatment can reverse the symptoms. Hypoglycemia, especially if accompanied by seizures, may result in a clinical picture compatible with CJD, and even MRI studies can show restricted diffusion in the cortex and basal ganglia but with relative sparing of the thalamus [137]. Hepatic encephalopathy can occur in both acute and chronic liver failure and manifest with a wide spectrum of neuropsychiatric symptoms, as well as movement disorders [138]. Especially, acute forms can show even overlapping MRI abnormalities, such as FLAIR and DWI cortical ribboning (usually sparing the perirolandic and occipital areas), but usually associated white matter hyperintensities in the internal capsule and subcortical white matter [139]. Rapid shifts in sodium can lead to extrapontine myelinolysis, presenting with encephalopathy and extrapyramidal syndromes, while the T2-weighted and DWI imaging may show hyperintensities in the basal ganglia, features resembling those seen in CJD [129], but these abnormalities usually disappear over weeks to months.

(E) A series of rare malignant conditions may pose serious challenges in the differential diagnosis of CJD. Primary CNS lymphoma, a diffuse B-cell non-Hodgkin’s lymphoma with an aggressive course and rising incidence [140], leads to a rapidly progressive cognitive decline, but the MRI shows usually enhancing lesions in contact with the cerebrospinal fluid [141]. Intravascular B-cell lymphoma is an even rarer condition and notoriously difficult to diagnose, leading to seizures, rapid cognitive decline, and upper and lower motor neuron symptoms. Again, imaging findings rather suggest a CNS vasculitis, with multiple T2/FLAIR hyperintensities in the white matter. Since the prominent neuronal damage may cause increases in 14-3-3 protein, RT-QuIC may help differentiate these cases from CJD, although the prognosis is significantly worse for the CNS lymphomas [142].

(F) Immune-mediated encephalitis. A series of antibody-mediated inflammatory disorders of the CNS, occurring either as paraneoplastic syndromes or as idiopathic conditions, may closely mimic CJD but respond very well to immune therapies. As such, every effort should be directed at identifying them [141]. While, in paraneoplastic syndromes, the antibodies are usually directed against intracellular antigens, more recently, a series of autoimmune encephalopathies with antibodies directed against cell-surface neuronal receptors or synaptic proteins [143] have been described, which are associated with underlying neoplasms in a variable proportion of cases. The onset is usually with psychiatric disturbances, but associated movement disorders (such as facial-brachial dystonic seizures) and the finding of CSF pleocytosis and MRI features suggestive of encephalitis, with a propensity for involvement of the limbic lobe, may aid in diagnosis [143]. Serum and CSF should be evaluated for antibody titers, although these may be initially negative in up to 50% of cases [144]. Table 2 provides an overview of the antibodies described in a series of autoimmune encephalopathies and their targets, as well as the incidence of underlying cancers and the most common neoplasia type.

#### 2.8.2. Imagistic Differential Diagnosis

Several other conditions may show resembling imagistic abnormalities, as shown in Table 3.

## 3. Therapeutic Attempts and Future Perspectives

Currently, there are no treatments approved for prion disease. Therapeutic strategies focus on maintaining quality of life and on symptomatic treatment.

Because sensory stimuli (light, noise, touch, or movement) may cause distress to patients with visual hallucinations or precipitate myoclonus, the patient may benefit from being placed in a quiet environment, with soft lighting, and the number of visitors should be limited [174].

Treatment of neuropsychiatric symptoms with benzodiazepines in patients already exhibiting ataxia may be a concern and place them at risk for falls; hence, selective serotonin or norepinephrine reuptake inhibitors (SSRIs and SNRIs) and trazodone should be preferred. In patients with extrapyramidal symptoms, typical antipsychotics should be avoided to minimize the risk of exacerbation. If hallucinations are distressing to the patient, an atypical antipsychotic (quetiapine) could be tried in low doses [174].

The caregiver burden should not be overlooked, as they are often worried by the genetic etiology, lack of education regarding transmissibility, and are unfamiliar with the disease progression [25,175].

### 3.1. Assessed Pharmacological Agents

A series of pharmacological agents have undergone formal clinical trials but with negative results.

- Flupirtine, a triaminopyridine shown in vitro to protect neurons from apoptosis caused by amyloid beta peptides and prion protein fragments, was evaluated in a study focusing on cognitive decline in patients with CJD. Although the rate of progression of dementia was slowed in the 13 patients randomized to flupirtine compared to the 15 patients randomized to the placebo, the overall survival rate was not influenced [25,176].
- Quinacrine (300 mg/day), an antimalarial drug that supposedly could prevent the conversion of PrP to disease-associated protein forms, was assessed in two clinical trials: an open-label trial in the UK (PRION1) and a double-blind, placebo-controlled, stratified-randomization treatment trial in the US [177]. Unfortunately, no difference in mortality rates could be demonstrated in either trial [178,179], despite a transient improvement in symptoms at the beginning of the treatment [180].
- Pentosan polysulfate has poor penetrance across the blood–brain barrier and had to be delivered via intraventricular injection. Although the survival was prolonged in observational studies, no symptomatic clinical benefit was discerned [181], and the aggressive delivery procedure was followed by many complications, mainly subdural effusions [182].
- Doxycycline, a tetracycline antibiotic with good blood–brain barrier penetration and shown to inhibit the aggregation of PrP proteins and reverse the protease resistance of PrP^Sc^ [183], had promising results in animal experiments [184], but in the clinical setting, it also failed to prolong survival [185], except when administered in the early stages [186].

### 3.2. Future Directions

Our increasing understanding of the disease at the molecular level has highlighted novel therapeutic directions. Considering the crucial role of the abnormally folded PrP^Sc^ in CJD pathogenesis, pharmacologically stabilizing the folded PrP^C^ and preventing its conversion to the disease-associated isoform could interfere with the pathogenic cascades [187]. 

#### 3.2.1. Active Immunization

Neuroinflammation has been increasingly shown to be involved in the pathogenesis of various neurodegenerative diseases [44,188,189], CJD included. As such, the possibility of immunization against CJD has been evaluated.

The conversion of PrP^C^ to PrP^Sc^ most likely occurs on the plasma membrane, and the misfolded protein enters the cytoplasm through endocytosis. T cells are activated only via simultaneous activation of the T-cell receptor (TCR) and CD28. To prevent excessive activation, T cells express CTLA-4 (cytotoxic T lymphocyte-associated antigen 4), which competes with CD28 in the process of T-cell activation, as does PD1 (programmed cell death 1). Prions promote CTLA-4 expression, thereby inducing immune tolerance [187].

To overcome immune tolerance, several approaches have been pursued:

- The administration of truncated, dimeric, or cross-linked PrP peptides has been shown to elicit strong immune responses in mice [190].
- DNA vaccines encoding specific PrP sequences can enhance the immune response [191], although their efficacy in preventing prion diseases is questionable [192].
- Using bacterial or viral vectors may bypass immune tolerance, as does mucosal vaccination [193].
- Finally, the identification of exposed regions of the misfolded protein allowed for the development of vaccines targeting these regions [194], with robust immune responses but, again, with less clear efficacy [195].

However, the widespread expression of PrP under physiological conditions and some toxicological concerns raised by these vaccines makes the overall applicability of these strategies questionable [25].

#### 3.2.2. Monoclonal Antibodies

Therapeutic strategies aiming at reducing the pathogenic PrP^Sc^ could lead to strain selection. Mice treated with quinacrine had transiently reduced levels of PrP^Sc^; after which, the latter recovered rapidly, possibly because the drug reduced certain subsets of conformers, but other prion protein conformations continued to spread [196].

The use of anti-PrP monoclonal antibodies was supported by both in vitro and in animal experiments [197,198], leading to a first clinical trial with an intravenous humanized monoclonal antibody to cellular prion protein (PRN100) in six CJD patients compared to historical untreated patients [199]. Although well tolerated and effective in clearing the abnormal protein (as shown by the report of the two autopsied cases), no influence on the clinical progression could be demonstrated [199], a scenario very similar to the results of the studies with the anti-Aβ antibodies lecanemab, donanemab, and aducanumab for Alzheimer’s disease [200]. Moreover, some anti-PrP antibodies proved neurotoxic due to the formation of a R208-H140 hydrogen bond (the H-latch) in the antibody molecule. Using PrP sequences unable to form this bond can neutralize the neurotoxicity [201].

#### 3.2.3. Immune Modulation

As shown before, a series of immune checkpoints, such as PD-1, CTLA-4, or LAG-3 (lymphocyte activation gene 3), are involved in inducing immune tolerance and prevent the body’s own immune system from clearing the abnormal protein. Immune checkpoint inhibitors are already used in certain subtypes of cancer [202], and attempts have been made to use them in CJD as well. However, neither a PD-1 nor LAG-3 blockade had a significant influence on PrP^Sc^ deposition or the disease course in animal experiments [203,204], suggesting that these molecules will not reproduce in neurodegenerative diseases the success seen in cancer [205].

#### 3.2.4. Gene Therapy

Gene therapy could be a promising alternative for carriers of the *PRNP* mutation, in which symptoms occur in late adulthood and early therapeutic intervention could delay (or even prevent) the progression of the disease.

Antisense oligonucleotides (ASOs) are single-stranded synthetic nucleotides that bind to a target mRNA and prevent the transcription of the target protein via RNase H degradation of the RNA–ASO complex [206]. ASOs directed against the *PRNP* gene administered intraventricularly in mouse models have been shown to reduce the deposition of PrP^Sc^ and extend survival [207] with no significant side effects. As such, prophylactic ASO treatment in carriers of the PRNP gene would be a viable alternative to prevent the onset of the disease [208].

Alternatively, RNA interference (RNAi) uses microRNAs (miRNAs), short interfering RNAs (siRNAs), or short hairpin RNAs (shRNAs) to “silence” the target gene [205]. Lentiviral-mediated RNAi was shown to reduce the expression of the pathogenic protein and prolong survival in mice [209].

PrP-Fc2 is a highly soluble and stable PrP dimer able to inhibit PrP^Sc^ replication. Lentiviral-mediated transfer of PrP-Fc2 to the brains of prion-infected mice has significantly extended survival rates, showing the potential of being developed as a prophylactic approach [210]. However, extensive research is still needed before translating these genetic therapies into clinical trials [187].

#### 3.2.5. Targeted Protein Degradation Therapies

Targeted protein degradation strategies are appealing in most neurodegenerative diseases with the accumulation of abnormal and misfolded proteins, such as Alzheimer’s disease, Parkinson’s disease, Huntington’s disease, and CJD. They use small molecules to increase ubiquitin–proteasome system-mediated protein degradation with proteasome-targeting chimeras (PROTACs) [211] or enhance lysosome-dependent protein degradation with lysosome-targeting chimeras (LYTACs) [212], autophagy-targeted chimeras (AUTACs) [213], or chaperone-mediated autophagy (CMA) [214]. Of the CMA-based degraders, the most studied is heat shock protein 70 (HSP70), which has been found to decrease with age, thereby increasing the risk for neurodegenerative diseases in the elderly [215]. The lack of HSP70 accelerates the progression of prion disease [216], while its overexpression has a neuroprotective effect [217]. However, issues relating to off-target effects, limited solubility and permeability across the blood–brain barrier, or metabolic instability must be improved before the clinical translation of these strategies [187].

#### 3.2.6. Stem Cell Therapies

Stem cells can be used to repair neurons damaged by the misfolded prions. They can be classified into embryonic stem cells (ESCs), mesenchymal stem cells (MSCs), induced pluripotent stem cells (iPSCs), and neuronal stem cells (NSCs) [218]. Intracerebral transplantation of fetal neural stem cells in animal models of prion disease prolonged both the incubation period and survival time [219], while nasal delivery of MSCs reduced neuroinflammation without impacting survival [220]. Despite exciting new developments in the area of stem cell research [221], clinical translation is still hampered by ethical issues and the risk of immune rejection and tumorigenesis [218].

## 4. Concluding Remarks: Challenges in the Quest for a Cure for Prion Diseases

Despite significant efforts in this area, there is currently no available therapy for prion diseases. As in most neurodegenerative diseases, patient studies miss the earliest stages of disease development, limiting opportunities for experimental interventions. In research on animal models, difficulties arise from an inability to recreate human-specific processes. The development of induced pluripotent stem cell (iPSC) technology has expanded the range of available human tissue models by enabling the design of three-dimensional structures (cerebral organoids) that can be modeled to reproduce the various subtypes of CJD [222].

Specifically, in prion disease, research is hampered by a series of factors, such as:

1. The low incidence of prion disease (about 1–2/1 million persons or 5/1 million persons aged over 65) poses serious difficulties in designing clinical trials. This could be overcome by optimizing the exchange of information between researchers and clinicians and sharing diagnostic tools and protocols, thereby facilitating data collection and recruitment of subjects [177].
2. The first clinical signs appear late in the disease course when degeneration is already quite advanced. In addition, the clinical presentation being unspecific, diagnosing CJD can be quite challenging and time-consuming, aided by the rapid, sometimes fulminant course to akinetic mutism and death in a few months.
3. We still lack specific markers that could be used as primary endpoints in clinical trials. To date, most of the trials were considered successful if they prolonged life, but the disease duration varies naturally in the various genetic subtypes. Moreover, given the expanding area of stem cell and organ/tissue transplantation, the need for testing donors with reliable markers for asymptomatic prion disease is even more stringent to avoid iatrogenic transmission.
4. From animal experiments, we know that a series of compound-resistant PrP^Sc^ strains can develop following the administration of a therapeutic compound [223]. Two hypotheses have tried to explain this phenomenon: (1) the “cloud” hypothesis, which posits that a prion strain contains a mixture of conformational variants from which the best-suited one in a specific environment thrives and becomes the dominant one while the others are progressively eliminated from the cloud over multiple propagation cycles [224], and (2) the “deformed templating” hypothesis, which postulates that prion conversion is not always fully faithful, leading to subsequent generations of prions with heterogeneous conformations. The new conformation(s) may possess a selective advantage in a specific environment and ultimately may become the dominant conformational variant [225]. As such, in vitro models must consist of cells capable of being infected by a wide range of PrP^Sc^ strains.

Nonetheless, although in the early stages, the novel therapeutic targets highlighted by research findings and the transition from externally administered drugs to recruiting immune cells to combat the disease show promise for the future. In our opinion, gene therapy (ASOs and RNA interference) would be applicable in patients with the familial form of CJD who can undergo genetic testing if a family member has been diagnosed with the disease, and vaccines could apply to persons with occupational exposure to prion diseases or potentially also to caregivers of patients with CJD [226], while sporadic cases would benefit from enhanced protein degradation strategies or intrabody approaches [131]. It is worth remembering that cancer therapies were barely effective half a century ago, whereas, today, their efficiency has increased significantly [187].

## Figures and Tables

**Table 1:** Epidemiological, clinical, and neuropathological characteristics of the subtypes of sporadic CJD (adapted from references [54,57,83].

Subtype of Sporadic CJD	Mean Age of Onset (Years)	Mean Disease Duration (Months)	Neuropathological Features	Characteristic Clinical Symptoms/Signs
MM(V)1	69	4	Spongiform changes in the cerebral (occipital) cortex, cerebellum, thalamus and striatum	Dementia, visual impairments, ataxia, myoclonus
VV2	65	6–9	Involvement of the cerebellum, basal ganglia, and diencephalon; spongiform changes often limited to the deep layers of the neocortex	Ataxia, with dementia following later in the disease course
MV2K	65	9–17	Amyloid kuru plaques in the cerebellum, involvement of the basal ganglia	Ataxia, extrapyramidal signs, dementia
MM2T	42–52	16–18	Atrophy of the medial thalamus and inferior olive, patchy spongiform changes in the cortex	Ataxia, double vision, sleep disturbances, psychiatric symptoms, followed by cognitive decline
MM2C	61–64	16–18	Rapidly progressive dementia	Spongiform changes and PrP deposits in the neocortex
VV1	42–44	18–21	Dementia, followed by ataxia, extrapyramidal signs	Spongiform changes in the neocortex, hippocampus, and striatum

**Table 2:** Characteristics of antibody-mediated autoimmune encephalopathies (adapted from ref. [143,145]).

Type of Antigen	Autoantibody Target	Incidence of Cognitive Impairment	Clinical Features	Possibility of Underlying Cancer and Type of More Common Neoplasia	MRI Features (T2/FLAIR)	Ref.
Surface antigens	AMPA receptors	100%	LE, hyponatremia	64%, small cell lung, thymoma	Hyperintensities in medial temporal lobes and/or cerebellum	[146]
	NMDA receptors	90–100%	LE, psychosis, facio-brachial dyskinesias	40–60%, usually ovarian teratoma	Normal aspect or non-specific regional changes	[147]
	DPPX	80–100%	Sleep disturbances, gastrointestinal symptoms (diarrhea)	10%, hematologic malignancies	Normal or non-specific changes	[148]
	GABA_B_ receptors	80–100%	LE, epileptic seizures	40–60%, thymoma, bronchial carcinoma	Hyperintense signals in medial temporal lobes	[149]
	GABA_A_ receptors	67%	Refractory seizures, status epilepticus	25–40%, thymoma	Hyperintense signals in multiple cortical and subcortical areas	[150]
	LGI1 receptors	90–100%	LE, facio-brachial dystonic seizures, myoclonus, hyponatremia,	10%, thymoma, bronchial carcinoma	Hyperintensities in medial temporal lobes and basal ganglia	[151]
	CASPR2	40–80%	LE, stiff person syndrome, ataxia	10–20%, thymoma	Normal aspect or hyperintensities in medial temporal lobes	[152]
	IgLON5	30–40%	Sleep disturbances, ataxia	rare	Normal aspect	[153]
	mGLUR1	rare	Cerebellar ataxia	Rare cases of Hodgkin’s lymphoma	Normal aspect or cerebellar atrophy	[154]
	mGLUR5	90%	LE, seizures	50%, Hodgkin’s lymphoma	Normal aspect or hyperintensities in various brain regions	[155]
	GlyRα1	40–50%	Stiff person syndrome, rigidity, myoclonus, seizures	10%, thymoma	Normal aspect or non-specific features	[156]
	Neurexin 3α	40–50%	Orofacial dyskinesias	unknown	Normal aspect	[157]
Intracellular antigens	AK5	100%	LE	Not cancer-associated	Temporal lobe hyperintensities	[158]
	amphiphysin	30%	LE, peripheral neuropathy	80%, small cell lung, breast	Normal aspect or temporal lobe hyperintensities	[159]
	ANNA1 (Hu)	10–20%	LE, cerebellar ataxia, sensory neuronopathy	80–90%, small cell lung carcinoma	Temporal lobe hyperintensities	[160]
	ANNA2 (Ri)	10–20%	Ataxia, opsoclonus-myoclonus	75%, small cell lung carcinoma, breast adenocarcinoma	Non-specific features	[161]
	ANNA3	10–20%	LE, cerebellar ataxia, peripheral neuropathy	80–90%, small cell lung carcinoma	Non-specific changes	[161]
	GAD	3–5%	LE, ataxia, seizures	8%, small cell lung carcinoma	Atrophy of temporal and frontal lobes	[162]
	GFAP	15–60%	Tremor, myoclonus, ataxia	35%, teratomas	Hyperintensities in the posterior parts of the thalamus	[163]
	ITPR1	20%	Cerebellar ataxia, seizures	30–40%, breast cancer	Normal aspect	[164]
	Ma2	60–70%	LE, sleep disorders, narcolepsy	90%, testicular neoplasias	Temporal lobe hyperintensities	[165]
	CRMP5 (CV2)	30%	LE, chorea, cerebellar ataxia, myelopathy, optic neuritis	90%, small cell lung carcinoma	Normal aspect or multiple hyperintensities in the temporal lobe, basal ganglia, thalamus and frontal lobe	[166]

Abbreviations: AK5—adenylate cyclase 5; AMPAR—α-amino-3-hydroxy-5-methyl- 4-isoxazole- propionic acid receptor; ANA—anti-nuclear antibody; ANNA—anti-neuronal nuclear antibody; CASPR2—contactin-associated protein 2; CRMP5—collapsing response-mediator protein-5. DPPX—dipeptidyl aminopeptidase-like protein 6; GABA_A_R, γ-aminobutyric acid type-A receptor; GABA_B_R, γ-aminobutyric acid type-B receptor; GAD—glutamic acid decarboxylase; GFAP—glial fibrillary acidic protein; GlyRα1, glycine receptor subunit alpha-1; IgLON5, immunoglobulin-like cell adhesion molecule IgLON family member 5; ITPR1—inositol trisphosphate receptor type 1; LE, limbic encephalitis; LGI1, leucine-rich glioma inactivated 1; mGluR1, metabotropic glutamate receptor 1; mGluR5, metabotropic glutamate receptor 5; NMDAR, N-methyl-D-aspartate receptor.

**Table 3:** Conditions with MRI features resembling those seen in CJD.

Main Brain Areas Involved	Condition	Similar Aspects	Differences	Supplemental Clues to Diagnosis	Ref.
Cortex	Severe hypoxic-ischemic encephalopathy	DWI, FLAIR and T2 hyperintensities in the cerebral cortex, hippocampus, basal ganglia	- Involvement of the perirolandic areas - swelling of the affected areas	Acute onset following cardio-respiratory arrest, asphyxia, drowning	[167]
	Autoimmune encephalopathy	DWI, FLAIR and T2 hyperintensities in the cortex, insula and cingulate areas	FLAIR/T2 hyperintensities involving mainly hippocampus, amygdala, mesial temporal lobe	Specific autoantibodies	[168]
	Infectious encephalitis (herpetic encephalitis)	Asymmetrical DWI and FLAIR/T2 hyperintensities in the cortex, mainly of the medial temporal lobe and orbitofrontal lobe	T1 hyperintensity in the presence of necrosis and hemorrhage	Acute onset, fever, stiff neck	[169]
	Postictal state following focal or generalized seizures	DWI, FLAIR/T2 hyperintensities in the hippocampus, neocortex, splenium of the corpus callosum, basal ganglia and thalami	Abnormalities are transient and disappear on subsequent imaging studies		[170]
	Hyperammonemia	Extensive cortical signal abnormalities with restricted diffusion affecting mainly cingulate gyrus and insula	Involvement of the perirolandic cortex	Increased serum ammonia	[171]
Basal ganglia	Extrapontine osmotic demyelination	Bilateral symmetrical FLAIR/T2 hyperintensities in the globus pallidus, putamen, thalamus	Absent DWI abnormalities	Rapid correction of hypo- or hyperosmolar states	[167]
	Ebstein Barr virus encephalitis	DWI/FLAIR/T2 hyperintensities in basal ganglia, cortex, and splenium of the corpus callosum	Abnormalities are transient		[169]
	Autosomal dominant striatal degeneration	DWI/FLAIR/T2 signal abnormalities in the striatum	- Sparing of globus pallidus and thalamus - characteristic antero-posterior gradient in the putamen	Familial clustering	[172]
Thalamus	Variant CJD	Pulvinar and double hockey stick signs	Lack of cortical involvement	Younger age of onset	[173]
	Wernicke encephalopathy	FLAIR/T2 hyperintensities in the medial thalamus	- Cortical involvement rare - signal abnormalities also in the periaqueductal region and mammillary bodies	Presence of conditions leading to thiamine deficiency	[113]

## Data Availability

No new data were generated during the writing of this manuscript.
